# Vitamin D deficiency and risk of recurrent aphthous stomatitis: updated meta-analysis with trial sequential analysis

**DOI:** 10.3389/fnut.2023.1132191

**Published:** 2023-06-22

**Authors:** Sadeq Ali Al-Maweri, Gamilah Al-Qadhi, Esam Halboub, Nader Alaizari, Asma Almeslet, Kamran Ali, Safa A. Azim Osman

**Affiliations:** ^1^College of Dental Medicine, QU Health, Qatar University, Doha, Qatar; ^2^Department of Basic Dental Sciences, Faculty of Dentistry, University of Science and Technology, Sana'a, Yemen; ^3^Department of Maxillofacial Surgery and Diagnostic Sciences, College of Dentistry, Jazan University, Jizan, Saudi Arabia; ^4^Department of Oral Medicine, Oral Pathology and Oral Radiology, Faculty of Dentistry, Sana'a University, Sana'a, Yemen; ^5^Vision College in Riyadh, Riyadh, Saudi Arabia; ^6^Department of Oral Maxillofacial Surgery and Diagnostic Sciences, College of Dentistry, Riyadh Elm University, Riyadh, Saudi Arabia

**Keywords:** aphthous stomatitis, vitamin D, association, risk factor, meta-analyses

## Abstract

**Background:**

Growing evidence suggests a significant association between vitamin D deficiency and RAS. Hence, the present meta-analysis and trial sequential analysis sought to investigate the potential association between low serum vitamin D levels and RAS.

**Methods:**

PubMed, Scopus, Embase, and Web of Science were comprehensively searched on December 1^st^, 2022 to retrieve all relevant studies. The grey literature was also searched via ProQuest. All case-control studies on the association between vitamin D and RAS were considered. The quality appraisal of the included studies was done using Newcastle-Ottawa scale. RevMan 5.0 and trial sequential analysis (TSA) programs were used for analyses.

**Results:**

A total of 14 case-control studies with 1468 subjects (721 RAS patients and 747 controls) were included. The pooled data revealed a significant association between low serum levels of vitamin D and the risk of RAS (mean difference = – 8.73, 95% CI: – 12.02 to – 5.44, I^2^ = 94%, P < 0.00001). Additionally, TSA findings indicated that the current studies surpassed the required information size, confirming that the differences were reliable.

**Conclusion:**

The available evidence suggests that Vitamin D deficiency may have a role in the pathogenesis of RAS. Therefore, evaluation of vitamin D should be considered in RAS patients. Additionally, the results support the possibility of using vitamin D supplements in the management of RAS patients with inadequate serum levels of vitamin D. Future interventional studies are required to evaluate the benefits of vitamin D replacement in prevention and treatment of RAS.

## Introduction

Recurrent aphthous stomatitis (RAS) - also known as recurrent aphthous ulcers or canker sores - is the commonest cause of oral mucosal ulceration (1–3). RAS is a highly prevalent condition affecting up to 25% of the general population, mainly adolescent and young adults, although it can occur at any age (3, 4). It is characterized by recurring, painful, ovoid or round, single or multiple ulcers of the oral mucosa, and primarily affect the non-keratinized mucosa (3, 4). The RAS-associated pain and discomfort might be severe and impact the patients' quality of life adversely by interfering with routine oral functions such as eating, swallowing, and speaking (1, 3, 5). Clinically, there are three variants of RAS: minor (less than 1 cm in diameter), major (more than 1 cm), and herpetiform (2–3 mm across) (2–4). Minor RAS is the most common form accounting for 90% of all RAS cases (1, 3).

Despite the extensive research done on the topic, the exact etiopathogenesis of RAS remains unclear (1). An immunological reaction to an unknown trigger is considered the most plausible mechanism involved in the development of RAS (6, 7). Several systemic and local factors increase predisposition to RAS including psychological stress, genetic makeup, immunological dysfunction, mucosal trauma, gastrointestinal disorders, hematological factors, and nutritional and hematinic deficiencies (1, 3, 6–10). The potential role of nutritional deficiencies of certain vitamins and minerals has been explored extensively in the literature (10). In this regard, numerous studies assessed hematinic and vitamins deficiencies (such as, B-complex vitamins and Folic acid) in RAS patients. However, the results remain inconclusive (11–16).

In recent years, the role of vitamin D in pathogenesis of several oral diseases including RAS has generated a significant level of interest (12, 14, 17, 18). Vitamin D, a lipid soluble secosteroid, plays key biological roles in calcium-phosphorus homeostasis and bone metabolism (17, 19). Recent evidence supports the role of vitamin D in inhibition of inflammatory process: Vitamin D is believed to modulate the immune system through inhibition of maturation of dendritic cells, and establishing a balance between different components of the immune system (17, 20, 21). In regard to systemic health, vitamin D deficiency has been linked to many disorders including hypertension, musculoskeletal disorders, obesity, cancers and autoimmune diseases (17, 22–26). In relation to oral health, a growing body of evidence links vitamin D deficiency to several oral mucosal diseases such as oral lichen planus and RAS (27, 28). In context of the latter, many recent studies investigated the potential association between vitamin D and the risk of RAS but showed inconsistent results (11–14, 28–30). Our previous meta-analysis, which involved all relevant studies published up to June 2019 (n = 5) revealed a significant association between vitamin D deficiency and RAS (31). Since then, numerous case-control studies have investigated the role of vitamin D in RAS, and appeared to report variable results (11–14, 32–35). Hence, we sought to update the available evidence regarding the potential association between low serum levels of vitamin D and RAS, supported by a trial sequential analysis (TSA). TSA is a novel approach used in systematic reviews and meta-analysis to control the random errors in the conventional meta-analysis and identifies the information size and weather further studies are still required or not (36).

The focused research question for this study was: “Is low serum level of vitamin D associated with RAS?

## Methods

The present meta-analysis followed PRISMA 2020 guidelines and PICO/PECO principles (37), and the protocol was registered in PROSPERO (ID: CRD42022365428).

### Eligibility criteria

All case-control and cohort studies that investigated the association between serum levels of vitamin D (25-hydroxycholecalciferol) and RAS, and fulfilled the following criteria were considered eligible: (1) comprised systemically healthy RAS subjects who were compared with systematically healthy control subjects, (2) the outcome measures reported serum levels of vitamin D quantitatively (mean ± SD).

The exclusion criteria were: (1) Lack of control group, (2) experimental studies, (3) case reports (4) case series, (5) missing or inadequate quantitative data (means of vitamin D), (6) editorials, and (7) review papers.

### Search strategy

Two investigators independently conducted extensive online searches on December 1, 2022 in PubMed, Scopus, Embase, and Web of Science databases for all relevant studies from date of inception till and including November 2022. The grey literature was also searched via Proquest. The following Mesh terms and free keywords were used for the electronic searches: “Stomatitis, Aphthous”[Mesh] (for PubMed) OR “recurrent aphthous ulcers” OR “aphthous ulcers” OR “recurrent aphthous stomatitis” OR “recurrent aphthosis” OR “recurrent oral ulcer” Or “aphthous stomatitis” AND “vitamin D” OR “25-hydroxycholecalciferol” (Detailed search strategy is presented in Supplementary Table 1). The online searches were supplemented with a manual search of the references of the included studies. The retrieved studies were then exported to EndNote program, and duplicates were removed.

### Data extraction

Two investigators (NA, GA) independently extracted all relevant data using a pre-designed form. The following data were extracted: authors, year of publication, country, study design, sample size, age of participants, the means and SD of serum levels of Vitamin D (ng/mL).

### Assessment of quality of evidence

Two investigators (NA, GA) independently assessed the quality of the included studies using the Newcastle-Ottawa Scale (NOS). The quality of each study was evaluated based on the following three domains: selection of cases and controls; comparability of cases and controls; and assessment of the exposure. Subsequently, each study was judged as either high quality (at least 7 stars); moderate quality (4–6 stars); or poor quality (0–3 stars).

### Data synthesis

Statistical analyses were conducted using Review Manager (RevMan) Version 5.3 (Copenhagen: The Nordic Cochrane Centre, the Cochrane Collaboration, 2014). The mean difference (MD) in vitamin D between the two groups along with 95% confidence interval (CI) were calculated. The heterogeneity across the included studies was evaluated using the Cochrane Q test (χ2 test) and I-squared index (*I*^2^). A P-value of < 0.05 was considered statistically significant.

### Trial sequential analysis (TSA)

TSA software version 0.9.5.10 beta was used for TSA (www.ctu.dk/tsa). In brief, we used two-sided trial sequential monitoring boundary type, and the required information size (RIS) was estimated (36, 38).

### Publication bias

Publication bias was assessed using funnel plot and Egger's test.

## Results

### Study selection

**Figure 1:**
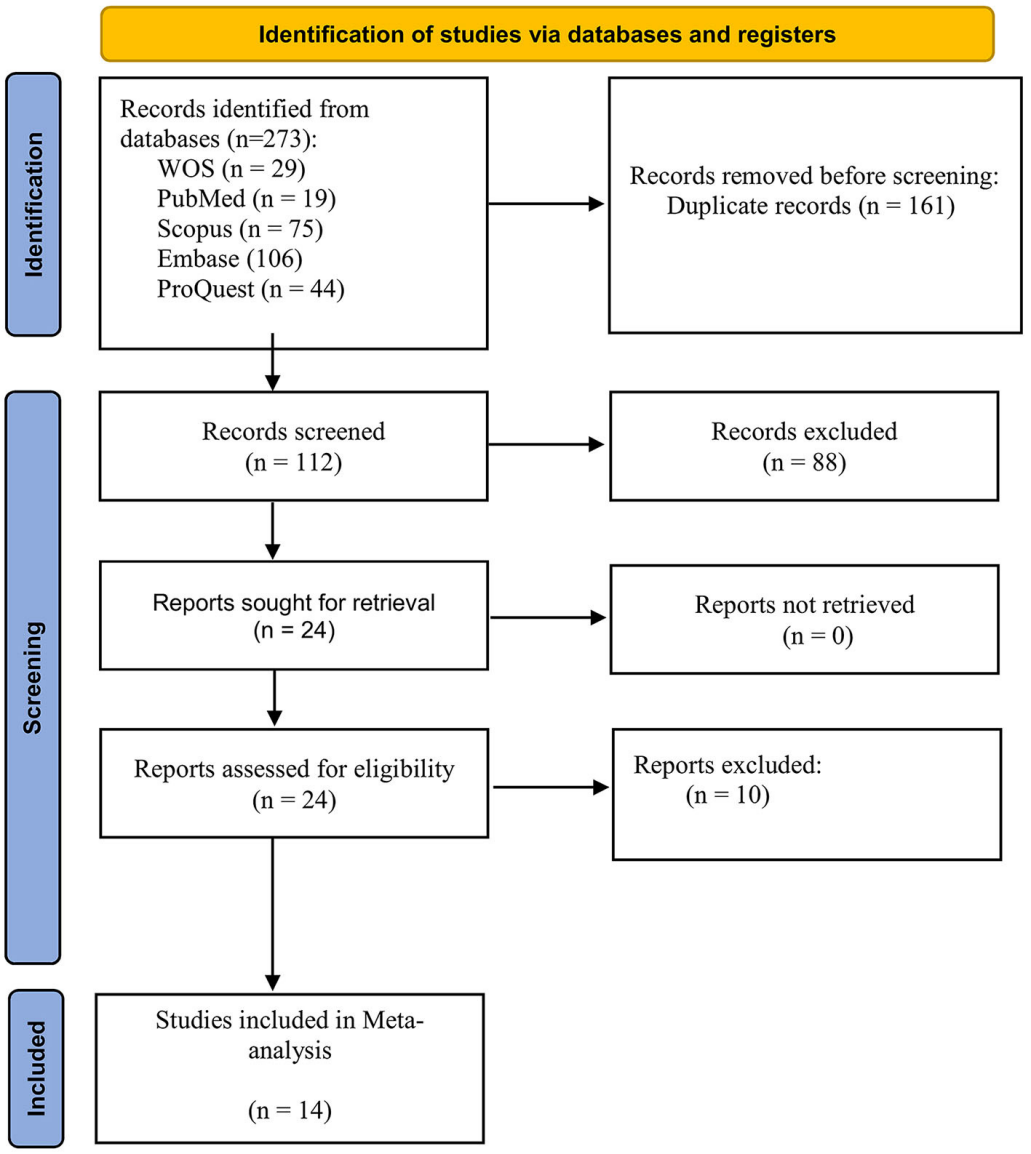
Flowchart of the study search strategy.

A total of 273 records were retrieved from online searches, 161 of which were duplicates and were thus removed (Figure 1). The titles and abstracts of the remaining 112 articles were screened by two independent investigators (SA, GA) for inclusion. Of these, 88 articles were found to be irrelevant and were excluded. The full text of the potentially eligible 24 articles were read by the two investigators, and 10 were excluded for various reasons (Supplementary Table 2). Eventually, 14 studies were included in the present meta-analysis.

### General characteristics of the included studies

A total of 14 case-controlled studies comprising 1468 subjects (721 RAS patients and 747 controls) were included in this meta-analysis (11–14, 18, 28–30, 32–35, 39, 40). Six studies were conducted in Turkey (12–14, 34, 39, 40), three in Iran (28, 30, 35), one in Poland (18), one in India (33), one in Saudi Arabia (32), one in the United Arab Emirates (11) and one in Iraq (29). The mean age of study participants ranged from 29.26 to 40.60 years, and from 27.44 to 40.80 years in the control groups. Two studies were conducted in children with mean age ranging from 7.6 to 8.7 years (34, 40). Of 721 RAS cases, around 56% (*n* = 404) were females (Table 1). Eight studies (28–30, 32, 34, 35, 39, 40) included patients with minor RAS, two studies (18, 33) included patients with minor, major, and herpetiform RAS, and one study (12) included patients with minor and major RAS, while three studies (11, 13, 14) did not mention the type of RAS. All the included studies assessed the serum levels of vitamin D, seven of which used ELISA (enzyme-linked immunosorbent assay) (29, 30, 32–35, 40), while four studies (11, 18, 28, 39) used ECLIA (electro-chemiluminescence binding assay). Three studies (12–14) did not mention the type of the assay. With respect to diagnostic criteria of RAS, all studies relied on clinical presentation and history of recurrence of RAS, while one study did not provide sufficient information (14) (Table 1).

**Table 1:** General characteristics of the included studies.

**Author and year**	**country**	**Study design**	**Participants No/gender/mean age (years)**		**Type of RAS**	**Diagnostic criteria of RAS**	**Assay method**
			**RAS**	**Controls**			
Koparal et al. (12)	Turkey	Case-control	N:70 F:37, M:33 Age: 40.60	N:70 F:34, M:36 Age:40.31	Minor, Major	Clinical, history of recurrence RAS within ≥ 2-year	NS
Mustafi et al. (33)	India	Case-control	N: 40 F: 18, M:22 Age: 34.32	N:40 F:18, M:22 Age: 33.43	Minor, Major, Herpetiform	Clinical, history of RAS minimum 3 episodes within last 3 months	ELISA
Oner et al. (41)	Turkey	Case-control	N: 60 F:34, M:26 Age: 31	N: 70 F: 41, M: 29 Age: 36.1	NS	Clinical, history of RAS > 3 times/year	NS
Zakeri et al. (35)	Iran	Case-control	N:43 F:32, M:11 Age:32.56	N:43 F:35, M:8 Age: 33.74	Minor	Clinical, history of RAS at least 3 periods/year	ELISA
Al-Amad and Hasan (11)	UAE	Case-control	N:52 F:20, M:32 Age:34	N:52 F:20, M:32 Age:31	NS	Clinical, History of recurrence of similar ulcers	ECLIA
Hussein et al. (32)	KSA	Case-control	N:70 F:39, M:31 Age:29.26	N:70 F:33, M:37 Age:32.59	Minor	Clinical, history of minimum 3 ulcers/year	ELISA
Nalbantoglu and Nalbantoglu (34)	Turkey	Case-control	N:72 F:39, M:33 Age: 8.7	N:70 F:34, M:36 Age: 7.6	Minor	Clinical, history of RAS minimum 3 episodes within last year	EIA
Tamer and Avci (14)	Turkey	Case-control	N:20 F:15, M:5 Age:34	N:20 F:14, M:6 Age:33.9	NS	Medical records	NS
Ali (29)	Iraq	Case-control	N: 30 F:30, M:0 Age: 36.4	N: 30 F: 30,M: 0 Age: 33.6	Minor	Clinical, history of RAS at least 3 times/year	ELISA
Bahramian et al. (28)	Iran	Case-control	N: 26 F10, M:16 Age:38.8	N: 26 F:9, M:17 Age: 40.80	Minor	Clinical, history of RAS at least 3 times/year	ECLIA
Oztekin and Oztekin (39)	Turkey	Case-control	N: 40 F:25, M:15 Age: 31.2	N: 70 F: 38, M:32 Age: 27.44	Minor	Clinical, history of RAS at least 3 times/year	ECLIA
Krawiecka et al. (18)	Poland	Case-control	N: 66 F:42, M: 24 Age: 34.15	N: 66 F:50, M: 16 Age: 32.05	Minor, Major, herpetiform	Clinical, history of regular recurrence of ulcers	ECLIA
Khabbazi et al. (30)	Iran	Case-control	N: 46 F:18, M: 28 Age: 33.4	N: 49 F: 19, M: 30 Age: 34.1	Minor	Clinical, history of at least 3 episodes per year.	ELISA
Başarslan and Kaba (40)	Turkey	Case-control	N: 86 F:45, M: 41 Age: 8.61	N: 71 F:31, M: 40 Age: 8.06	Minor	Clinically and history	ELISA

RAS, recurrent aphthous stomatitis; M, male; F, female; NS, Not Specified; ECLIA, electro-chemiluminescence binding assay; ELISA, enzyme-linked immunosorbent assay; EIA, Enzyme immunoassay.

Concerning the outcome measures, all studies assessed and compared serum levels of vitamin D in RAS and controls, and eight studies also (11, 13, 18, 29, 30, 32, 34, 39) assessed the association between serum levels of vitamin D and RAS variables such as duration, severity, and frequency (Table 2).

**Table 2:** Summary of the main outcomes.

	**Vitamin D levels (ng/ml)**		**Conclusion**
	**RAS**	**Controls**	
Koparal et al. (12)	22.16 ± 9.55	26.15 ± 11.01	Vitamin D levels were significantly lower in RAS patients compared to controls (*P =* 0.019)
Mustafi et al. (33)	14.34 ± 6.73	26.23 ± 3.99	Vitamin D levels were significantly lower in RAS patients compared to control (*P* < 0.0001)
Oner et al. (41)	12.42 ± 2.8	16.95 ± 4.10	Although RAS had lower serum vitamin D levels than controls, no statistically significant difference was found between the groups (*P =* 0.056). Also, there was no significant association between vitamin D levels and duration or frequency of RAS (P>0.05).
Zakeri et al. (35)	13.89 ± 8.19	22.59 ± 16.06	Vitamin D level in control group was significantly higher than in the case group (*P =* 0.002)
Al-Amad and Hasan (11)	53.6 ± 24.6	51.5 ± 26.9	No significant difference was found between RAS patients and healthy controls (*P =* 0.68). However, binary logistic regression showed a significant association between vitamin D deficiency and number of RAS (*P =* 0.027)
Hussein et al. (32)	20.25 ± 6.01	29.92 ± 6.80	The mean level of vitamin D in RAS group was significantly lower than the control group (*P < * 0.001). The results also showed a significant correlation between vitamin D deficiency and number, frequency and severity of RAS (*P* < 0.0001)
Nalbantoglu and Nalbantoglu (34)	16.4 ± 8.6	23.1 ± 11.5	Vitamin D levels were significantly lower in RAS patients compared to control group (*P =* 0.002). There was no significant correlation between serum vitamin D levels and number, frequency, healing time and severity of RAS
Tamer and Avci (14)	13.6 ± 6.5	20.9 ± 10	The mean serum vitamin D level was significantly lower in RAS patients compared to healthy individuals (*P =* 0.01)
Ali (29)	13.90 ± 12.72	22.08 ± 17.77	Vitamin D levels were significantly lower in RAS group (*P =* 0.045). There was a significant correlation between the serum levels of 25(OH) D and the number RAS in each attack (*r* = 0.435; *P =* 0.016). However, no significant correlation was found between serum Vitamin D levels and duration and frequency of RAS
Bahramian et al. (28)	33.07 ± 12.41	50.89 ± 9.30	RAS group showed significantly lower vitamin D levels than control group (*P =* 0.001)
Oztekin and Oztekin (39)	11.00 ± 7.03	16.4 ± 10.19	RAS group showed significantly lower vitamin D levels (*P =* 0.004). Yet, no significant association was observed between vitamin D levels and RAS size, healing time and frequency
Krawiecka et al. (18)	16.81 ± 8.45	19.22 ± 10.44	Although vitamin D levels were lower in RAS patients, the results were not statistically significant (*P =* 0.207). The lowest vitamin D level was observed in the most severe form of RAS (based on frequency of RAS), but the results did not reach statistical significance (*P =* 0.074)
Khabbazi et al. (30)	12.10 ± 7.70	27.40 ± 9.70	RAS group showed significantly lower vitamin D levels than control group (*P =* 0.001). Yet, no correlation was found between vitamin D levels, duration, number of ulcers and frequency of RAS
Başarslan and Kaba (40)	12 ± 4.53	31 ± 7	Individuals with RAS revealed significantly lower vitamin D levels than healthy control group (*P =* 0.001)

RAS, recurrent aphthous stomatitis.

### Quality of the included studies

A summary of the quality assessment of the included studies is presented in Table 3. Of the included studies, 12 (12–14, 18, 28–30, 32–35, 40) were of moderate quality, while two studies (11, 39) were of high quality.

**Table 3:** Quality of studies assessed by Newcastle Ottawa Scale (case-control studies).

**Study**	**Selection**	**Comparability**	**Exposure**	**Total score**	**Quality**
Koparal et al. (12)	^**^	^**^	^*^	5	Moderate
Mustafi et al. (33)	^***^		^**^	5	Moderate
Oner et al. (41)	^**^	^**^	^**^	6	Moderate
Zakeri et al. (35)	^*^	^**^	^**^	5	Moderate
Al-Amad and Hasan (11)	^****^	^**^	^**^	8	High
Hussein et al. (32)	^**^	^**^	^**^	6	Moderate
Nalbantoglu and Nalbantoglu (34)	^**^	^*^	^**^	5	Moderate
Tamer and Avci (14)	^**^		^**^	4	Moderate
Ali (29)	^***^	^*^	^**^	6	Moderate
Bahramian et al. (28)	^***^	^*^	^**^	6	Moderate
Oztekin and Oztekin (39)	^**^	^**^	^**^	7	High
Krawiecka et al. (18)	^***^	^*^	^**^	6	Moderate
Khabbazi et al. (30)	^***^	^*^	^**^	6	Moderate
Başarslan and Kaba (40)	^***^	^*^	^**^	6	Moderate

### Qualitative results

Of the 14 included studies, 11 studies (12, 14, 28–30, 32–35, 39, 40) found significantly lower serum levels of vitamin D in RAS patients compared to the controls (Table 2); one study found insignificant (*P* = 0.056) lower serum levels of vitamin D in RAS patients (13); while two studies did not find any differences between the two groups (11, 18).

Eight studies (11, 13, 18, 29, 30, 32, 34, 39) assessed the association between serum levels of vitamin D and RAS variables such as duration, severity, and frequency. Five studies (13, 18, 30, 34, 39) found no significant association between vitamin D and duration, frequency, and severity of RAS, while three studies showed a significant association between serum levels of vitamin D and number of RAS (11, 29, 32) (Table 2).

### Meta-analysis results

**Figure 2:**
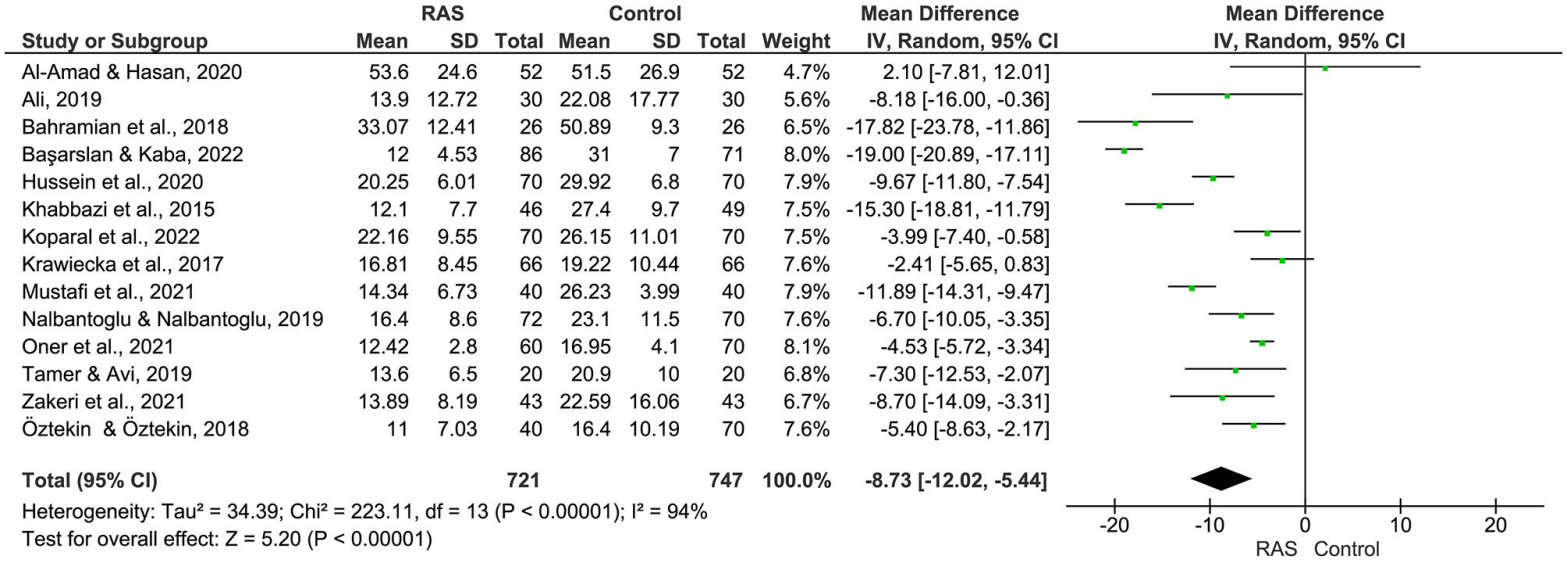
Meta analysis of vitamin D levels between RAS and controls.

The pooled results of the 14 studies revealed a significant association between low serum levels of vitamin D and the risk of RAS (MD = – 8.73, 95% CI: – 12.02to – 5.44, *I*^2^ = 94%, *P* < 0.00001 (Figure 2).

### TSA results

**Figure 3:**
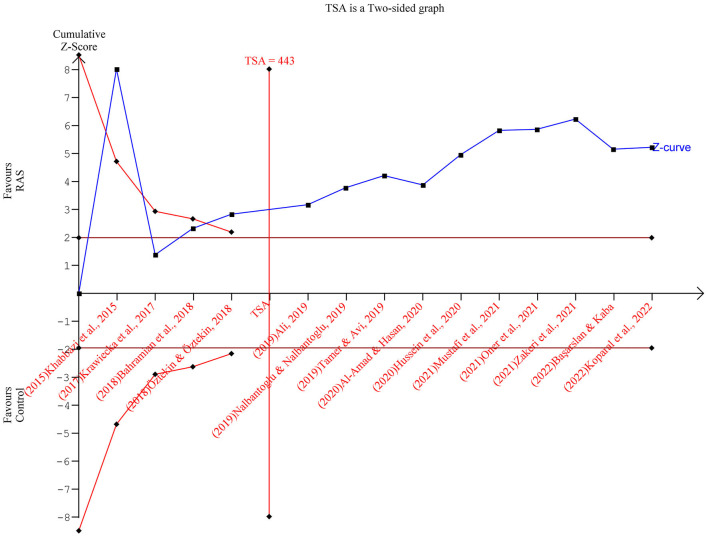
Trial sequential analysis results.

Figure 3 depicts the TSA: The cumulative Z curves crossed the conventional boundary and the trial sequential monitoring boundary and surpassed the required information size (*n* = 443) as well. As such, the evidence is reliable and confirmatory, and further trials are no longer needed.

### Publication bias

**Figure 4:**
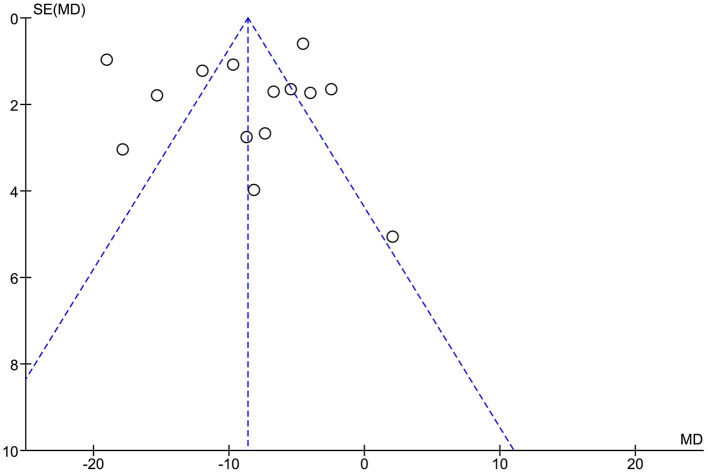
Funnel plots.

The funnel plot (Figure 4) reveals symmetric distribution of the included studies, indicating no publication bias.

## Discussion

As discussed earlier, RAS is a common mucosal disease that may impact adversely on the patients' quality of life (5). Despite a large body of research on the topic, the exact etiopathogenesis of RAS remains unclear (1, 6, 10). Given the lack of a specific etiology, RAS management is challenging (2, 42–44). In light of the mounting evidence regarding the potential role of vitamin D in the pathogenesis of certain oral mucosal diseases including RAS (13, 29, 31, 35), the present meta-analysis was conducted to answer the following focused question: Is low serum levels of vitamin D associated with higher risk of RAS? Overall, the results of the pooled 14 studies revealed a significant association between low serum levels of vitamin D and the risk of RAS. Additionally, the result of the meta-analysis was supported by the TSA findings, which showed that the current studies surpassed the required information size, confirming that the differences were reliable. However, the qualitative analysis revealed conflicting results regarding the association between serum levels of vitamin D and severity and frequency of RAS.

The results of the present updated meta-analysis confirm our original meta-analysis (31), and substantiate many previous systematic reviews and meta-analyses that found significant associations between vitamin D deficiency and several autoimmune diseases and dermatological conditions such as lupus erythematosus, vitiligo, autoimmune bullous dermatoses, rheumatoid arthritis, and primary Sjögren's syndrome (22–26). Interestingly, our results are in accord with a recent clinical trial which investigated the efficacy of vitamin D supplementations in RAS patients with vitamin D deficiency, and reported a significant reduction in the frequency of RAS episodes, number of ulcers, and duration of healing time after one year (45). The exact mechanism behind the effects of vitamin D on RAS is still unclear yet, but may be explained by its immunomodulatory effects. Studies confirmed that vitamin D has strong immunomodulatory effects on both innate and acquired immunity responses, as well as on cytokines levels (20, 46, 47), all of which are thought to be involved in the pathogenesis of RAS (6, 7, 10).

It is recognized that the level of any evidence obtained from each meta-analysis is largely dependent on the quality of the included studies. Hence, we meticulously scrutinized the quality of all included studies using NOS, a reliable and validated appraisal tool. The results showed that two studies were of high quality (low risk of bias), and 11 were of moderate quality, and no study was with low quality, suggesting fair evidence. Additionally, the result of the meta-analysis was further substantiated by TSA results, which further confirmed the reliability and conclusiveness of the results (36).

The present updated meta-analysis has some limitations that should be considered. The main limitation is the marked heterogeneity across the included studies in terms of geography, age of the participants, methods of vitamin D ascertainment, types of RAS included, among others. This in turn may have biased the results. Additionally, although the included studies were conducted in different parts of the world and involved large samples (721 RAS cases and 747 controls), five studies (around 40% of the included studies) came from one country, Turkey, and thus the generalization of the results may not be appropriate.

In conclusion, the present updated meta-analysis confirms the association between low serum levels of vitamin D and the risk of RAS. Hence, vitamin D assessment may be considered in RAS patients. The results also support the use of vitamin D supplementations in RAS patients with inadequate serum levels of vitamin D. However, future interventional studies (for prevention and/or treatment purposes) investigating the effect of vitamin D supplements on RAS patients are required.

## Data availability statement

The original contributions presented in the study are included in the article/Supplementary material, further inquiries can be directed to the corresponding author.

## Author contributions

SA: study concept, search strategy, and drafting the manuscript. GA-Q: data extraction, quality appraisal, and drafting the manuscript. EH: concept of the study and critically revised and edited the paper. NA: data extraction, quality appraisal, and drafting the manuscript. AA: concept of the study, data analysis, and critically revised and edited the paper. KA: concept of the study and critically revised and edited the paper. SO: data curation and critically revised and edited the paper. All authors approved the final version.
