# Supplementary material for: Vitamin D deficiency and risk of recurrent aphthous stomatitis: updated meta-analysis with trial sequential analysis
Source: Front Nutr. 2023 Jun 22;10:1132191. doi: 10.3389/fnut.2023.1132191 (PMC10325032; doi:10.3389/fnut.2023.1132191)
Supplement: Supplementary file 1 [file Data_Sheet_1.docx]

| Supplementary Table 1. Databases: Applied search strategy, and numbers of retrieved studies  . | | | |
| --- | --- | --- | --- |
| Databases | Search strategy used | | Hits |
| MEDLINE searched via PubMed searched on  December 01, 2022, via www.ncbi .nlm.nih.gov/sites | | #1 Search ("vitamin D"[All Fields] OR "25-hydroxycholecalciferol"[All Fields]) AND ("stomatitis, aphthous"[MeSH Terms] OR "recurrent aphthous ulcers"[All Fields] OR "aphthous ulcers"[All Fields] OR "recurrent aphthous stomatitis"[All Fields] OR "recurrent aphthosis"[All Fields] OR "recurrent oral ulcer"[All Fields] OR “aphthous stomatitis” "[All Fields] ) | 19 |
| ISI web of science Core Collection was searched via web of knowledge on December 01, 2022, via apps.webofknowledge.com | # 1 search (“recurrent aphthous ulcers” OR “aphthous ulcers” OR “recurrent aphthous stomatitis” OR ”recurrent aphthosis” OR “recurrent oral ulcer” OR “aphthous stomatitis” (All Fields) 1946  # 2 search ALL fields (“vitamin D” OR “25-hydroxycholecalciferol” ))  2693  #3 search #1 AND #2 29 | | 29 |
|  |  | |  |
| Scopus searched via Scopus on December 01, via https://www.scopus.com | Search # 1 TITLE-ABS-KEY ( "recurrent aphthous ulcers"  OR  "aphthous ulcers"  OR  "recurrent aphthous stomatitis"  OR  "recurrent aphthosis"  OR  "recurrent oral ulcer"  OR  "aphthous stomatitis" )  7825  Search # 2 TITLE-ABS-KEY ( "vitamin D"  OR  "25-hydroxycholecalciferol" ) 136271  #3 search #1 AND #2 75 | | 75 |
| Embase was searched via on December 01, 2022https://www.embase.com/search/quick | ('aphthous stomatitis'/exp OR 'aphthosis oris' OR 'aphthous fever' OR 'aphthous stomatitis' OR 'aphthous ulcer' OR 'aphthous ulceration' OR 'aphtous stomatitis' OR 'aphtous ulcer' OR 'recurrent aphthous stomatitis' OR 'recurrent aphthous ulceration' OR 'stomatitis aphthosa' OR 'stomatitis, aphthous') AND ('vitamin d'/exp OR 'vitamin d') | | 106 |
| ProQuest was searched on December 01, 2022, via  https://www.proquest.com/?accountid=13370 | # search ("aphthous stomatitis" OR "recurrent aphthous ulcer" OR “aphthous ulcer”) AND ("vitamin D" (dissertations and theses) 44 | | 44 |
|  | 926 | |  |
| Total |  | | 273 |

**Supplementary Table 2: List of excluded studies and the reason of exclusion**

| # | **Reference** | **Reason for exclusion** |
| --- | --- | --- |
| **1** | Hussein FF, Sadek HS, Elmarssafy LH. Impact of Vitamin D3 Supplementation on Recurrent Aphthous Ulcer. Indian Journal of Pharmaceutical Sciences. 2022 Mar 1:255-65. | No control group |
| **2** | Susanto H, Kandarwati P, Budiarti S, Bramantoro T. The association between vitamin D/25 (OH) D and reproductive hormone in young women with recurrent aphthous stomatitis: An observational study. Journal of International Oral Health. 2022 Jan 22;12(4). | No control group |
| **3** | Xu K, Zhou C, Huang F, Duan N, Wang Y, Zheng L, Wang X, Wang W. Relationship between dietary factors and recurrent aphthous stomatitis in China: a cross-sectional study. Journal of International Medical Research. 2021 May;49(5):03000605211017724. | Vitamin D was not investigated |
| **4** | Hernández-Olivos R, Muñoz M, Núñez E, Camargo-Ayala PA, Garcia-Huidobro J, Pereira A, Nachtigall FM, Santos LS, Rivera C. Salivary proteome of aphthous stomatitis reveals the participation of vitamin metabolism, nutrients, and bacteria. Scientific reports. 2021 Aug 2;11(1):1-5. | Vitamin D was not investigated |
| **5** | Wang Z, Cao H, Xiong J, Lu Y, Deng Y, Nan H, Zheng S, Ye H, Cao Z. Recent advances in the aetiology of recurrent aphthous stomatitis (RAS). Postgraduate Medical Journal. 2022 Jan 1;98(1155):57-66. | review |
| **6** | Bazrafshani MR, Hajeer AH, Ollier WE, Thornhill MH. Recurrent aphthous stomatitis and gene polymorphisms for the inflammatory markers TNF-alpha, TNF-beta and the vitamin D receptor: no association detected. Oral Dis. 2002;8(6):303-7. | Irrelevant outcome |
| **7** | Slebioda Z, Szponar E, Dorocka-Bobkowska B. Vitamin D and Its Relevance in the Etiopathogenesis of Oral Cavity Diseases. Arch Immunol Ther Exp (Warsz). 2016;64(5):385-97. | Irrelevant outcome |
| **8** | Bolat M, Chiriac MI, Trandafir L, Ciubara A, Diaconescu S. ORAL MANIFESTATIONS OF NURITIONAL DISEASES IN CHILDREN. Romanian Journal of Oral Rehabilitation. 2016;8(2):56-60. | review |
| **9** | Bahali AG, Kokturk A, Guvenc U. Sociodemographic and clinical characteristics of patients with recurrent aphthous stomatitis. Turkderm-Turkish Archives of Dermatology and Venerology. 2014;48(4):242-8. | Irrelevant outcome |
| **10** | Koybasi S, Parlak AH, Serin E, Yilmaz F, Serin D. Recurrent aphthous stomatitis: investigation of possible etiologic factors. American Journal of Otolaryngology. 2006;27(4):229-32. | Irrelevant outcome |
